# Upregulation of follistatin and low apoptotic activity in intraductal oncocytic papillary neoplasm of the pancreatobiliary system

**DOI:** 10.1038/s41598-020-64920-6

**Published:** 2020-05-18

**Authors:** Jun Nakahodo, Yuki Fukumura, Tsuyoshi Saito, Kenichi Hirabayashi, Reiko Doi, Takuo Hayashi, Takashi Yao

**Affiliations:** 1 Department of Human Pathology, Juntendo University School of Medicine, Tokyo, Japan; 2 Department of Pathology, Tokai University School of Medicine, Kanagawa, Japan

**Keywords:** Gastroenterology, Oncology

## Abstract

Intraductal oncocytic papillary neoplasm (IOPN) is a rare intraductal tumor of the pancreatobiliary system. Currently, little is known about its distinct characteristics, unlike intraductal papillary mucinous neoplasms (IPMN) and intraductal papillary neoplasms of the bile duct (IPNB). The present study compared 22 IOPNs (18 pancreatic and 4 biliary) with those of 61 IPMNs/8 IPNBs. IOPNs were classified into pure and combined types, depending on the coexistence of IPMN/IPNB. Multiple gene expression analysis (nCounter system) was performed, and hierarchical clustering analysis separated IOPNs(n = 4) and IPMNs(n = 3)/ IPNBs(n = 3), and pathway score analysis supported the result. Volcano plot identified follistatin (FST) as the most upregulated mRNA in IOPN in comparison to the gastric subtype (log2 fold change of 5.34) and the intestinal subtype (that of 5.81) of IPMN/IPNB. The expression of FST in IOPN was also high in quantitative polymerase chain reaction and immunohistochemical analysis. We also found lower apoptotic activity in IOPN, particularly in pure type, compared to high-grade or invasive IPMN/IPNB using immunohistochemistry for cleaved caspase 3. But, combined type IOPN was more similar to IPMN/IPNB than pure IOPN. In conclusion, we proved that IOPN, particularly pure IOPN, is distinct from IPMN/IPNB in FST mRNA overexpression and exhibits lower apoptotic activity.

## Introduction

Intraductal oncocytic papillary tumor (IOPN) is a histologically unique tumor of the pancreatobiliary system wherein oncocytic, mitochondria-rich tumor cells grow intraductally, forming complicated, arborizing papillae^1–5^. IOPNs of the pancreas and the bile ducts are characteristically similar and have been considered as counterpart tumors^3–8^. Similar to intraductal papillary mucinous neoplasm (IPMN) and intraductal papillary neoplasm of the bile duct (IPNB), IOPNs in these sites are characterized by intraductal papillae with delicate fibrovascular cores and frequent mucin hypersecretion. Moreover, IOPN components are sometimes seen as a part of IPMN or IPNB. Hence, IOPN has been classified as a histological subtype of IPMN/IPNB until recently^2^. However, recent molecular studies have indicated that the characteristics of IOPN are significantly different from those of IPMN; hence, a recently published WHO classification considers pancreatic IOPN pathologically different from IPMN^1,9–11^. IOPN usually lacks *KRAS*/*GNAS* mutations, which are now considered driver events in IPMN. However, there is insufficient knowledge pertaining to the difference between IOPN and IPMN/IPNB.

Hence, this study aims to identify specific characteristics of IOPN by performing multiple gene expression analysis/digital gene expression quantification. We also validated whether the most upregulated gene, follistatin (FST), is specific to IOPN. Because FST’s role includes the inhibition of TGF-β pathway and recent *in vitro* studies have revealed that FST inhibits apoptotic activity^12–16^, we further investigated TGF-β mRNA expression and apoptotic activity among IOPN and IPMN/IPNB cases.

## Results

### Clinicopathological data

A clinicopathological summary of studied cases is presented in Tables 1A,B, and the data of each IOPN specimen are shown in Supplementary Table 5. No statistically significant difference was found in terms of patients’ age/sex, tumor site, duct type, or tumor stage between IOPN and IPMN/IPNB, as well as pure and combined IOPN. All IOPN cases were pathologically diagnosed as high grade or more; hence, a statistically significant difference was found in the histological grade between IOPN and IPMN/IPNB samples.

**Table 1A:** Clinicopathological characteristics of IOPN, IPMN, and IPNB cases.

	IOPN^*ϕ*^	IPMN		IPNB		*P* value IOPN vs. IPMN/IPNB
		Gastric type	Intestinal type	Gastric type	Intestinal type	
**No. of cases**	22	41	20	4	4	
**Age**						0.416
Mean	66.7	70.46	66.3	66.0	72.8	
Range	44–82	34–83	34–78	59–71	65–79	
**Gender**						0.303
Male	12	26	16	2	2	
Female	10	15	4	2	2	
**Tumor site**						0.235
Head	10	28	12	NA	NA	
Body and tail	8	10	7	NA	NA	
Head to Tail	0	3	1	NA	NA	
Intrahepatic bile duct	4	NA	NA	3	2	
Perihilar bile duct	0	NA	NA	1	2	
Distal bile duct	0	NA	NA	0	0	
**Duct type**						0.163
Main duct	0	6	3	NA	NA	
Branch duct	8	14	3	NA	NA	
Combined duct	10	21	14	NA	NA	
**Tumor size**						0.061
Mean	39.2	35.29	35.9	31.8	23.0	
Range	17–100	8–120	16–75	15–65	15–28	
**Histological grade**						0.003
Low grade	0	23	1	0	0	
High grade (no invasion)	12	7	7	2	3	
Invasion	10	11	12	2	1	
**T factor**						
Tis	12	7	7	2	3	
T1 (Perihilar bile duct)	NA	NA	NA	1	0	
T1a	8	2	3	0	1	
T1b	0	0	0	0	0	
T1c	1	1	0	0	0	
T2	0	5	8	1	0	
T3	1	3	1	0	0	
**N factor**						0.525
N0	21	15	18	2	4	
N1 or more	1	3	1	0	0	
**Stage**						
0	12	7	7	4	3	
I (Perihilar bile duct)	NA	NA	NA	1	0	
IA	9	2	3	0	1	
IB	0	5	8	0	0	
II (Perihilar bile duct)	NA	NA	NA	1	0	
IIA	0	1	0	0	0	
IIB	1	2	0	0	0	
III, IV	0	1	1	0	0	
**Concomitant other subtype**						0.264
None	15	33	14	4	4	
Gastric	5	—	6	—	0	
Intestinal	2	6	—	0	—	
Pancreatobiliary	0	2	0	0	0	
**B Clinicopathological characteristics of pure type and combined type of IOPN**.						
	**IOPN**	**IOPN**				**P value Pure vs. Combined**
		**pure**		**combined**		
		**p-IOPN**	**b-IOPN**	**p-IOPN**	**b-IOPN**	
**No. of cases**	22	12	3	6	1	
**Gender**		0.652				
Male	12	6	3	3	0	
Female	10	6	0	3	1	
**Tumor site**		0.896				
Head	10	7	NA	3	NA	
Body and tail	8	5	NA	3	NA	
Head to Tail	0	0	NA	0	NA	
Intrahepatic bile duct	4	NA	3	NA	1	
Perihilar bile duct	0	NA	0	NA	0	
Distal bile duct	0	NA	0	NA	0	
**Duct type**		0.502				
Main duct	0	0	NA	0	NA	
Branch duct	8	6	NA	2	NA	
Combined duct	10	6	NA	4	NA	
**Tumor size**		0.669				
Mean	39.2	39.9	30.7	37.7	65.0	
Range	17–100	17–100	25–37	20–70	65.0	
**Histological grade**		0.867				
Low grade	0	0	0	0	0	
High grade (no invasion)	12	6	2	3	1	
Invasion	10	6	1	3	0	
**T factor**						
Tis	12	6	2	3	1	
T1 (Perihilar bile duct)	NA	NA	NA	NA	NA	
T1a	8	5	1	2	0	
T1b	0	0	0	0	0	
T1c	1	1	0	0	0	
T2	0	0	0	0	0	
T3	1	0	0	1	0	
**N factor**		0.134				
N0	21	12	3	5	1	
N1 or more	1	0	0	1	0	
**Stage**						
0	12	6	2	3	1	
I (Perihilar bile duct)	NA	NA	NA	NA	NA	
IA	9	6	1	2	0	
IB	0	0	0	0	0	
II (Perihilar bile duct)	NA	NA	NA	NA	0	
IIA	0	0	0	0	0	
IIB	1	0	0	1	0	
III, IV	0	0	0	0	0	
**Concomitant other subtype**						
None	15	12	3	0	0	
Gastric	5	0	0	4	1	
Intestinal	2	0	0	2	0	
Pancreatobiliary	0	0	0	0	0	

^ϕ^p-IOPN, IOPN of pancreas; b-IOPN, IOPN of bile duct.

### Multiple gene expression analysis

**Figure 1:**
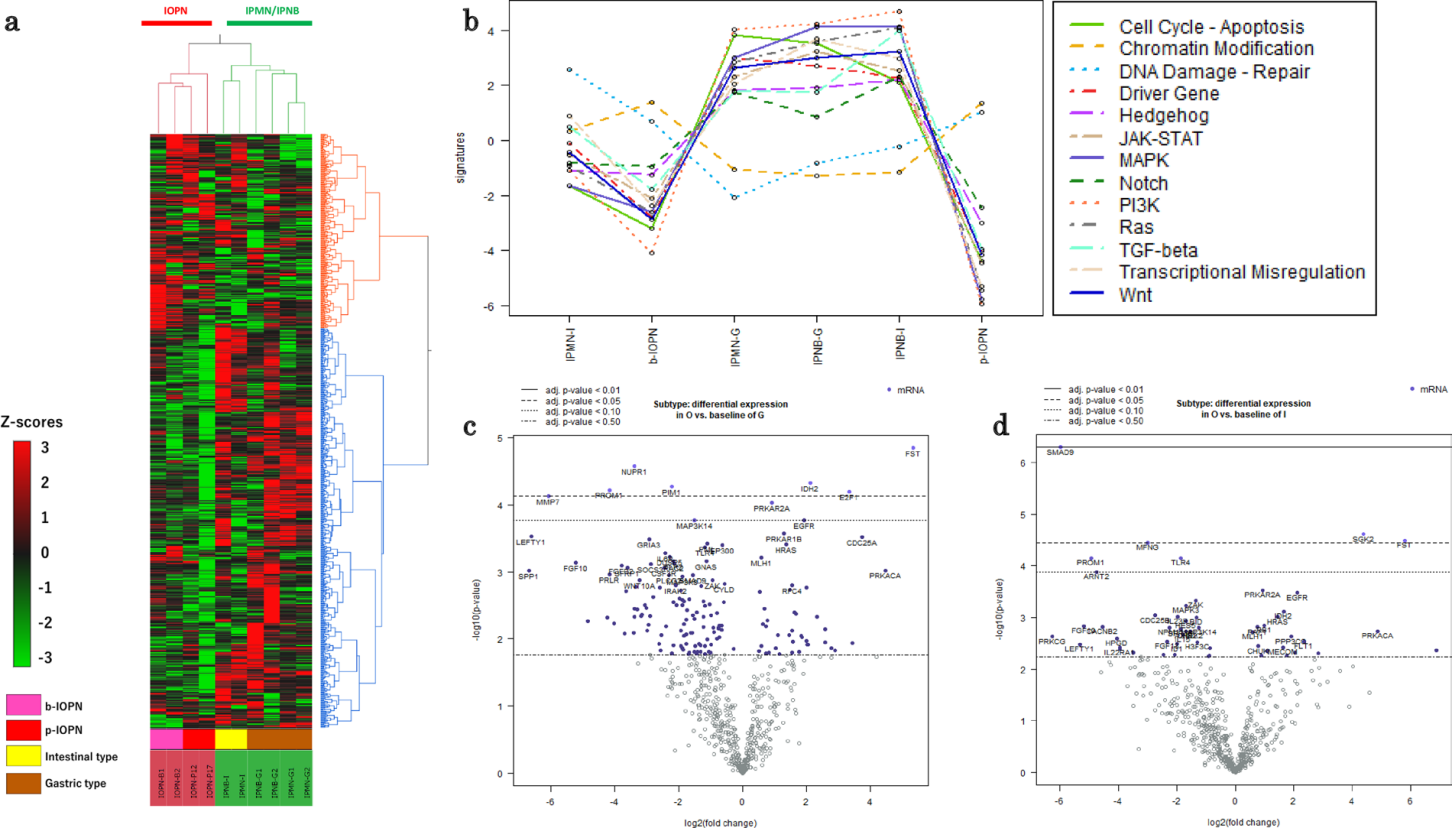
(**a**) Hierarchical cluster analysis based on expression of 770 genes. The heat map shows that IOPN specimens (n = 4) and IPMN (n = 3)/IPNB (n = 3) specimens are separated in cluster. The color scale for the heat map is shown in the lower left corner. IOPN, intraductal papillary oncocytic neoplasm; IPMN, intraductal papillary mucinous neoplasm; IPNB, intraductal papillary neoplasm of the bile duct. (**b**) Pathway score analysis of 13 canonical cancer pathways. Among the 13 pathways, 11 pathways were downregulated in IOPN specimens of the bile duct (n = 2) and the pancreas (n = 2). The pathway “Chromatin modification” was upregulated in IOPN specimens on the both sites compared to others. The pathway “DNA damage-repair” was upregulated IOPN on the both sites than in IPMN/IPNB specimens of gastric subtype and IPNB specimens of intestinal subtype, but not IPMN specimens of intestinal subtype. Higher Y axis value (signature) mostly corresponds to mostly increasing expression (specifically, each pathway score has positive weights for at least half of its genes). (**c**) Volcano plot between IOPN and gastric IPMN/IPNB. There were 5 relatively upregulated/5 downregulated genes with statistical significance (adjusted *p* < 0.10). FST was most significantly differentially upregulated in IOPN with the highest fold change (adjusted *p* < 0.05). Genes in the right and left halves of the graph were upregulated and downregulated in IOPN compared to gastric IPMN/IPNB. [X axis, log2 (fold change), Y axis, -log10 (*p*-value), dotted lines in the graph indicate various *p*-value thresholds.] (**d**) Volcano plot between IOPN and intestinal IPMN/IPNB. There were 2 relatively upregulated/5 downregulated genes with statistical significance (adjusted *p* < 0.10). Among the significantly upregulated genes, FST showed the highest fold change. Genes in the right and left halves of the graph were upregulated and downregulated ones in IOPN compared to intestinal IPMN/IPNB.

Hierarchical clustering analysis of the normalized data indicated a distinct cluster for 4 pancreatobiliary IOPN specimens, whereas IPMN and IPNB specimens comprised another cluster (Fig. 1a). To characterize the effect of altered gene expression, pathway analysis was performed, wherein IOPN of both the pancreas and bile ducts showed lower activity in 11 of the 13 pathways investigated. These 11 pathways are shown in Fig. 1b. “Chromatin modification” was found to be more activated in IOPN samples as compared with IPMN/IPNB samples. The overall appearance of pathway analyses indicated that IOPN and IPMN/IPNB were structured in a different pattern of activated/inactivated pathways (Fig. 1b).

From volcano plot analysis, several candidate genes were identified that could function as IOPN-specific biomarkers among the pancreatobiliary intraductal tumor samples, as shown in Fig. 1c,d and Supplementary Tables 6 and 7. Among the upregulated candidate genes, FST showed the highest log2 fold change (5.34 for the gastric subtype IPMN/IPNB sample with an adjusted *p*-value of <0.05 and 5.81 for the intestinal IPMN/IPNB sample with an adjusted *p*-value of <0.05), as shown in Supplementary Tables 6 and 7. Comparing IOPN of the pancreas and of the bile ducts, volcano plot analysis found no differentially expressed genes in a statistically significant manner (Supplementary Table 8, Supplementary Fig. 1).

### **qPCR of FST and TGF-**β **1**

**Figure 2:**
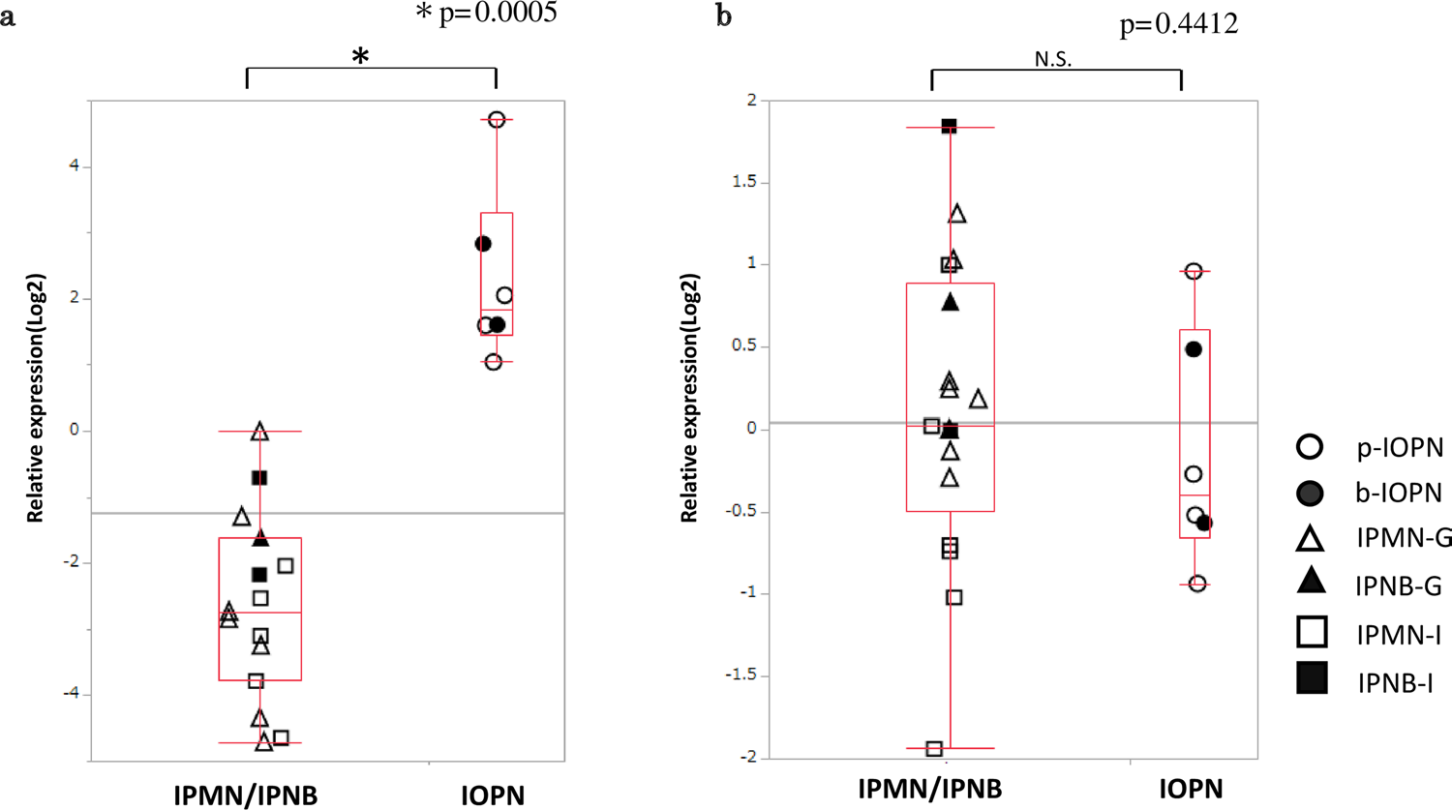
Quantification of FST-mRNA and TGF-β 1-mRNA (Results of qPCR). IOPN showed significantly higher expression of FST compared to IPMN/IPNB (**a**), whereas no significant difference was found in TGF-β 1 expression (**b**). [Y axis, log2 value of relative quantification of FST/TGF-βmRNA].

qPCR showed that IOPN tissue samples expressed more FST-mRNA compared with gastric/intestinal IPMN/IPNB tissue samples (*p* = 0.0005), as shown in Fig. 2a and consistent with the NanoString data results. There was no significant difference in terms of TGF-β1 expression between the samples (*p* = 0.4412). See Fig. 2b.

### Immunohistochemistry for FST and CC3

**Figure 3:**
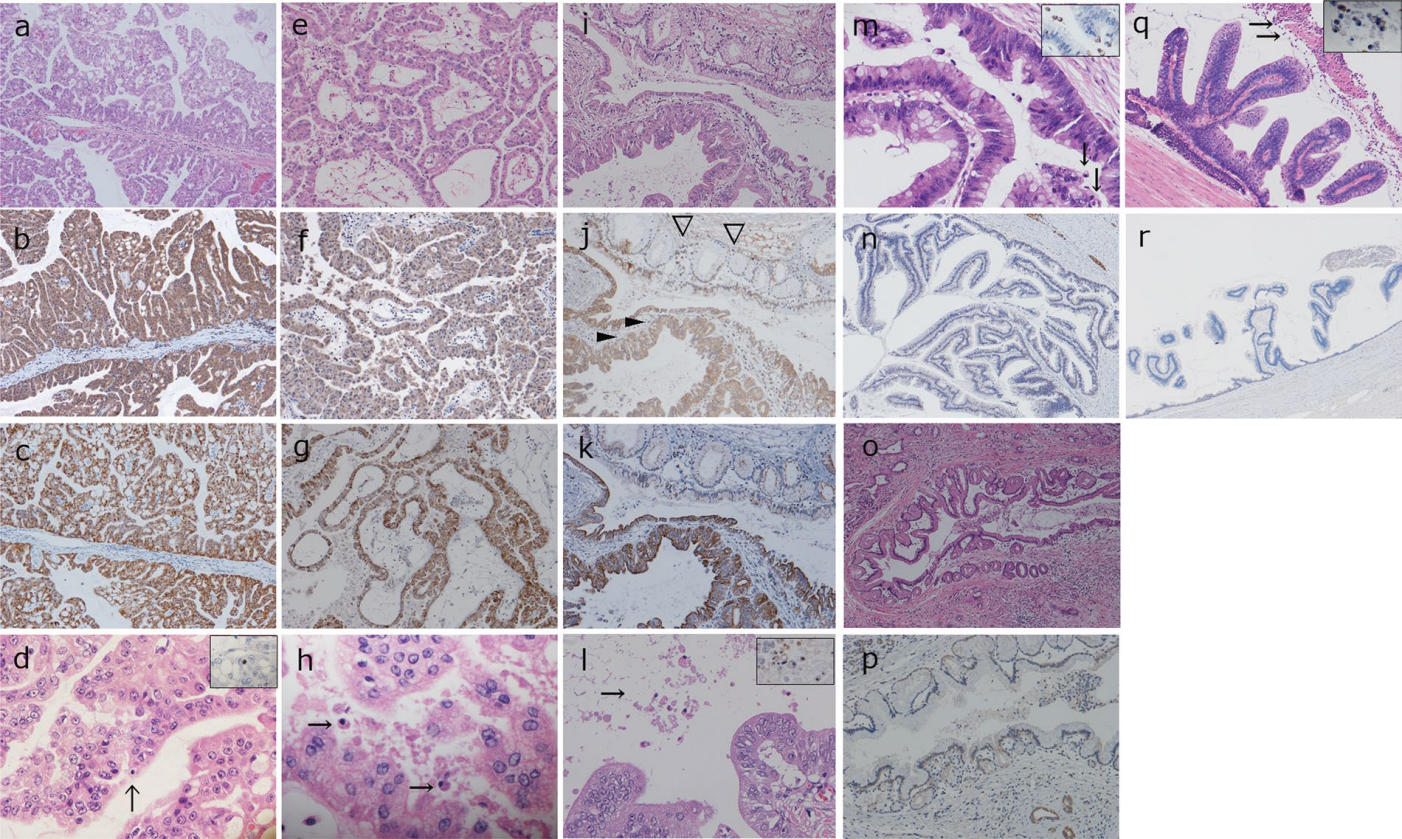
Representative histology and immunohistochemistry of IOPN and IPMN/IPNB. IOPN of pancreas, pure type (**a–d**), IOPN of bile duct, pure type (**e–h**), IOPN of pancreas, combined type (**i–l**), IPMN, intestinal subtype (**m,n**), IPMN, gastric subtype (**o,p**), and IPNB, intestinal subtype (**q,r**). Immunohistochemistry for FST (b, f, j, n, p, r), for mitochondria (c, g, k). Immunohistochemistry for Cleaved Caspase 3 (CC3) is shown in the inset of d, l, m, q. Intense/weak/negative staining for FST was seen in b, f /j / n, p, r. Note that the IOPN area is weakly positive for FST (white arrowheads), whereas gastric IPMN area is negative for FST (black arrowheads) in the combined type. Apoptotic figures were seen sparsely scattered/in aggregates in d, h, o/l, m, q (Arrows).

**Figure 4:**
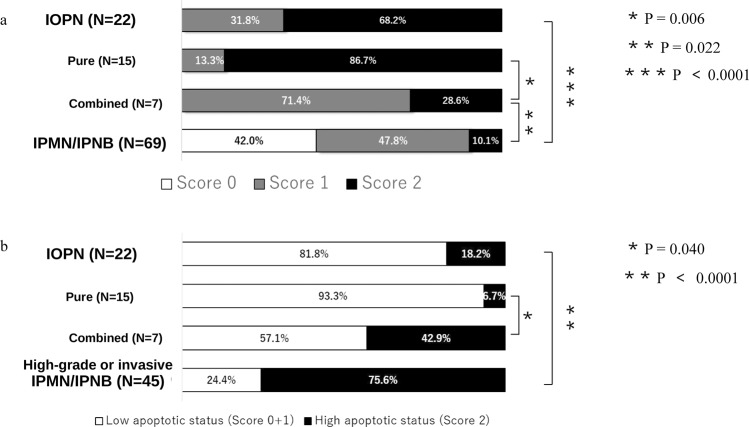
Summary of immunohistochemical results. (**a**) Distribution of FST scores in IOPN and IPMN/IPNB. FST scores showed significant difference between IOPN and IPMN/IPNB (*p* < 0.0001), and between pure and combined IOPN (*p* < 0.05, *p* = 0.006). (**b**) Distribution of Cleaved Caspase 3 (CC3) scores in IOPN and high-grade or invasive IPMN/IPNB. CC3 scores were significantly different between pure and combined IOPN (*p* < 0.05, *p* = 0.040), and IOPN and IPMN/IPNB samples (*p* < 0.0001).

Raw immunohistochemical data are provided in Supplementary Table 9. Representative microscopic figures of IOPN and IPMN/IPNB tissue samples are shown in Fig. 3. All IOPN samples including combined IOPNs were either weakly or intensely positive for FST, whereas FST expression was negative in 42.0%, weakly positive in 47.8%, and intensely positive in 10.1% of the IPMN/IPNB samples (Fig. 4a). In the FST-positive IPNB/IPMN cases (57.9%), tumor cells were positive for FST focally, not entirely, and FST-positive cells tended to have slightly oncocytic cytoplasm (data not shown). The difference between the FST scores of IOPN and IPMN/IPNB samples was statistically significant (*p* < 0.0001), as shown in Fig. 4a.

In combined IOPN cases, immunohistochemistry for FST was different between the IOPN area and other IPMN/IPNB components; IOPN components were weakly or intensely positivity for FST, whereas other components were negative or weakly positive for FST (Fig. 3j,k). In IOPN, tumor cells were positive for FST almost entirely; the tumor cells in the complex papillary portion were more intense positive, whereas those in the flat portion tended to be weakly positive. Pure IOPN showed higher intensity scores for FST compared with combined IOPN with a statistical significance of *p* < *0.05* (*p* = 0.006), as seen in Fig. 4a.

Immunohistochemistry for CC3 yielded no positive cells or only scattered in 93.3% of pure IOPN, in 57.1% of combined IOPN, and 24.4% of high-grade or invasive IPMN/IPNB specimens. (Fig. 3d,h,l,m,o,q). The differences between the immunohistochemically determined CC3 scores of IOPN and high-grade or invasive IPMN/IPNB samples and those between pure IOPN and combined IOPN samples were statistically significant, i.e., *p* < 0.0001 and *p* < 0.05 (*p* = 0.040), respectively (Fig. 4b).

## Discussion

IOPN is a histologically unique tumor of the pancreatobiliary system, in which oncocytic tumor cells grow in the complicated arborizing papillae. IOPN of the pancreas was first introduced by Adsay NV *et al*. in 1996 as intraductal oncocytic papillary neoplasms^5^. IOPN of the bile duct was reported as hepatic intraductal oncocytic papillary carcinoma by Martin *et al*.^17^. Although several genetic studies have reported the molecular events in IPMN/IPNB, studies on the molecular events for IOPN are few. Basturk O *et al*. identified several specific mutational events in IOPN of the pancreas, such as *ARHGAP26*, *ASXL1*, *EPHA8*, and *ERBB*, but the frequency of each event is low, and there are still only limited molecular data for IOPN^18,19^.

Our IOPN cohort (n = 22) showed no statistical difference from IPMN/IOPN clinicopathology including patients’ age, gender, tumor size, and duct-type/tumor site for pancreatic IOPN and IPMN. On the other hand, based on hierarchical clustering analysis using 770 genes, IOPNs were grouped as an independent cluster and distinguished from IPMNs/IPNBs. Pathway score analysis also showed that pancreatobiliary IOPN has different pathway patterns from IPMN/IPNB samples. Current knowledge regarding the differences between pancreatic IOPN and IPMN includes better prognosis, less association of colloid carcinomas, immunohistochemical attitude for human hepatocyte and Pepsinogen-I, and different mutational profiles of IOPN^7,10,11,20,21^. Here, we showed that IOPN is also distinct in terms of cancer pathway activation.

Multiple gene expression analysis indicated that the FST mRNA was the most upregulated gene in IOPN compared with both gastric and intestinal IPMN/IPNB, and FST upregulation in IOPN was validated with qPCR/immunohistochemical analyses. All IOPN specimens (including IOPN area of combine IOPN specimens) were diffusely immunopositive for FST, where pure-type showed intense immunoreactivity for FST more often compared with combined-type with statistical significance. In IPMN/IPNB, tumor cells with slight oncocytic change sometimes showed focal/weak FST expression.

We also found that apoptotic activity was lower in pure IOPN compared with IPMN/IPNB and combined IOPN with statistical significances using immunohistochemistry for CC3. CC3 is one of the key enzymes responsible for activating the apoptotic pathway and the CC3 antibody detects endogenous levels of the large fragments of activated CC3. Hence, this antibody is used to detect apoptotic cells. At present, the reported functions of FST have included the inhibition of TGF-β pathway by binding to activin, which is a TGF-β superfamily member^13,22–26^, and protection against apoptosis in an activin-independent manner by neutralizing oxidative stress^14^ and/or via attenuation of rRNA synthesis by nuclear localization of FST^15^. Because we did not include the functional analysis of FST-utilizing IOPN cells, we cannot identify the cause of lower apoptotic activity in IOPN, but it is important to understand how IOPN obtains apoptotic resistances because many anticancer drugs currently used in clinical trials take advantage of apoptotic signaling pathways to trigger death in cancer cells^27^. Further studies are thus necessary to determine the function of FST and identify what triggers lower apoptotic activity in IOPN.

Currently, the TGF-β pathway is known to associate with the malignant transformation of IPMN. Mohri D *et al*., reported about BMP-phospho-SMAD1/5/8 activation in intestinal subtype of IPMN^28^, whereas Okabayashi *et al*., studied the TGF-β/SMAD4 signaling, particularly in branch-duct IPMNs^29^. Recently, Qiu *et al*., investigated the role of the activin pathway in IPMN tumorigenesis using LSL-KRAS(G12D) in Pdx1-Cre mice^30^. However, the relationship between the TGF-β pathway and IOPN has yet to be reported. The upregulation of FST, but not of TGF-β in IOPN, compared to IPMN/IPNB in the present study may suggest that the downregulation of activin pathway via FST, but not TGFβ-SMAD4 pathway, is related to IOPN formation. However, further investigation on downstream factors of activin pathways is necessary for confirmation.

Our IOPN cohort included 15 pure IOPNs and 7 combined IOPNs. In the latter, other concomitant IPMN components existed in a serial fashion to IOPN components. Although both types of IOPN components showed papillary structures with a lining of 2–5 layers of tumor cells of cuboidal to columnar eosinophilic granular cytoplasm and intraepithelial lumina histologically, hence satisfying the definition of IOPN per the WHO 2019 classification, these two types were different; the combined type showed more similar results to IPMN/IPNB samples than the pure type in terms of immunohistochemical FST intensity and apoptotic activity/CC3 positivity. Separate analysis of pure and combined IOPN in the future study is recommended.

The existence of focal FST-positive eosinophilic cells in IPMN/IPNB specimens and the slight difference in FST intensity between the flat and papillary portions in an IOPN specimen suggest that cytoplasmic FST accumulation is related not only to gene expression but also to cellular degeneration/eosinophilic change.

The limitation of our study was that our analyses did not include IPMN/IPNB of pancreatobiliary subtypes due to an insufficient number of cases in our archives. Data on FST expression, apoptotic status, upregulated or downregulated pathways are required for future studies, with enough samples of pancreatobiliary subtypes for analysis.

In conclusion, this study showed the differences in cancer pathway activation pattern, FST expression, and apoptotic rate between IOPN and IPMN/IPNB specimens. IOPN may be classified into pure and combined types because these two classes differ in terms of FST protein expression and apoptotic rate.

## Materials and methods

### Materials

Cases of twenty IOPNs of pancreatobiliary glands (18 pancreatic and 4 biliary IOPNs), 61 IPMNs, and 8 IPNBs were enrolled in this study. The diagnosis and subtyping of IPMNs/IPNBs were performed according to WHO classification^1^, where the tumor portion with the highest histological grade was used for histological subtyping. As for IOPN, the cases in which 30–100% of total tumorous area satisfied WHO classification of IOPN were included in this study, where those without any other IPMN/IPNB component were classified as pure type (n = 15), and those with IPMN/IPNB component of gastric and/or intestinal subtype were classified as combined subtype (n = 7). All IOPN cases came from surgical resections between April 1990 and March 2018 at the University Hospital of Juntendo and University Hospital of Tokai.

All IPMN/IPNB cases were surgically resected during the same period. IPMN/IPNB cases of gastric and intestinal subtypes were used, and those of pancreatobiliary subtypes were excluded in this study because only a few cases of these were found in our archives. Accordingly, 41 gastric/20 intestinal IPMNs and 4 gastric/4 intestinal IPNBs were included in the study. All pathological specimens were reviewed by JN, YF, and TS. This study was approved by the Ethics Committee of Juntendo University, Tokyo, Japan (#2013160 for IPMN and #2017115 for IPNB) on Oct. 2018, Nov. 2017, respectively, and was performed according to the Declaration of Helsinki. The informed consent was obtained from all subjects.

### Collection of clinicopathological data

Information on patients’ age/sex, tumor site and size, duct type (for pancreatic IOPN and IPMN), histological grade, tumor stage was collected, and the data for both IOPN and IPMN/IPNB cases and pure IOPN and combined IOPN cases were compared.

### Sample preparation

Formalin-fixed, paraffin-embedded (FFPE) tissue sections were prepared. Depending on the tumor size, one to four continuous FFPE tissue sections (5 µm, mounted on positively charged slides) were dissected, and the tumor tissue was collected into a 1.7 ml microcentrifuge tube using a sterile razor blade. In the cases of IOPN/IPMN/IPNB with invasive carcinomas, only the non-invasive tumor tissue was collected.

RNA was isolated from the samples by using RNeasy FFPE Kit (Qiagen, Hilden, Germany) according to the manufacturer’s instructions. Multiple gene expression analysis and quantitative polymerase chain reaction (qPCR) were performed. Thereafter, the extracted RNA samples were quantified using a Nanodrop 1000 spectrophotometer (Thermo Fisher Scientific, AL, USA).

### Multiple gene expression analysis

Four IOPNs (2 from pancreas, 2 from bile duct), 3 IPMN, and 3 IPNB specimens were used. As 12 samples are analyzable per increment of nCounter system, we selected 4 IOPNs, 4 IPMNs (2 gastric and 2 intestinal subtypes) and 4 IPNBs (2 gastric and 2 intestinal subtypes) for nCounter analyses. All 12 samples were obtained by surgical resection at the Juntendo University School of Medicine. Selection criteria included cases showing typical histology for each subtype, having enough tumor volume for the nCounter analysis, and containing no or a few necrotic or inflammatory cell aggregates. Finally, one specimen each from intestinal IPMN and intestinal IPNB was rejected by the nCounter system because of data quality, hence, the data was obtained for the remaining 10 specimens [Supplementary Table 1].

The nCounter system quantifies mRNA expression of each gene utilizing a digital “barcode” system, without gene amplification process. In this study, nCounter PanCancer Pathways Panel, which quantifies 770 genes, was utilized. Each of these genes is known to be involved in 13 canonical cancer pathways, such as MAPK, JAK-STAT, and Notch pathways. In this system, tumors can be classified by gene expression profiling; the activity of 13 canonical cancer pathways and associated driver genes are captured by a biology-guided, data-driven approach. (https://www.nanostring.com/download_file/view/2103/3807)

Purified RNAs (100 ng) obtained from each specimen were hybridized overnight using the PanCancer Pathway Code Set^31^ (NanoString Technologies, WA, USA) at 65 °C. Further purification and binding of the hybridized probes to the optical cartridge were performed on nCounter Prep Station, and the cartridge was scanned on nCounter Digital Analyzer. RCC files obtained from NanoString Digital Analyzer were imported into nSolver 2.6 software (NanoString Technologies, WA, USA) and were checked for data quality using the default quality check settings. A “barcode” was used to determine the mRNA level, after which background correction was performed by subtracting the “mean + 2 standard deviation” value of the negative controls from the raw counts and then the adjusted raw counts were normalized to the geometric mean housekeeping genes. Relative expression values were calculated by dividing the mean values of all the samples in the following study groups by the mean values of one benign gastric IPMN specimen (Case No. P-1). We set seven patterns of study groups, namely IOPN, IPMN, IPNB, p-IOPN, b-IOPN, g-IPMN/IPNB, and i-IPMN/IPNB. Bioinformatic and statistical analyses were performed using nSolver Analysis Software, ver.4.0.62 and the PanCancer Pathways Advanced Analysis module. Hierarchical clustering was performed using the former, and pathway analysis and volcano plot were performed with the latter. Pathway analysis was performed by condensing each sample’s gene expression profile to calculate a pathway score by first principal component analysis^32^. P-values were calculated using Student’s *t*-test.

### qPCR of FST and TGF-b

qPCR for FST and TGF-b was conducted to validate the results of multiple gene expression analysis. Data from 7 IOPNs (5 from pancreas, 2 from bile duct) were compared with 14 IPMNs (8 gastric/6 intestinal subtypes), and 3 IPNBs (1 gastric/2 intestinal subtypes) [Supplementary Table 2]. All specimens were selected from surgically resected cases at the first author’s institution after 2010 because of the limitations from the joint research agreement and to ensure the RNA quality. qPCR was performed as previously described^21^. The β-actin gene served as an endogenous control for the normalization of expression levels. Details of the probes used for qPCR are listed in Supplementary Table 3. cDNA from one of each IOPN organ, gastric IPMN, and intestinal IPMN specimens could not be amplified for the FST gene; hence, qPCR results for *FST* were obtained for 6 IOPN, 12 IPMN, and 3 IPNB specimens.

### Immunohistochemistry

For diagnostic assistance and for subtyping/grading of IOPN and IPMN/IPNB specimens, immunohistochemical analysis of MUCs, human hepatocyte, mitochondria, and MIB-1 were performed at the beginning of this study. Immunohistochemical analyses of FST and cleaved caspase-3 (CC3) were performed to validate the results of multiple gene expression analysis and evaluate apoptotic activities in IOPN and IPMN/IPNB tumor tissues. Details of the primary antibodies used in this study are summarized in Supplementary Table 4. The immunohistochemical results were reviewed by JN, YF, and TS, and scored/recorded as follows: MUC1, MUC2, MUC5AC, MUC6, mitochondria, and human hepatocytes were scored 0, 1, and 2 when no positive cells were present, less than 50% of tumor cells were positive, and ≧50% of tumor cells were positive, respectively. Ki-67 labeling index (LI) value was expressed in terms of percentage and determined using the MIB-1 antibody. FSTs were evaluated for cytoplasmic staining and were scored as 0, 1, and 2 when it is negative for tumor cells, weakly positive, and intensely positive, respectively. CC3 was scored as 0, 1, and 2, when almost no positive cells are found, positive cells were scattered, and positive cells were seen in aggregates, respectively. The anterior lobes of the pituitary and palatine tonsils were used as positive controls for FST and CC3, respectively. The FST scores were compared among the study groups, i.e., IOPN vs. IPMN/IPNB and pure IOPN vs. combined IOPN. To identify the apoptotic activities of the tumors, CC3 scores were compared with high-grade or invasive specimens from each study group. i.e., IOPN vs. IPMN/IPNB and pure IOPN vs. combined IOPN.

### Statistics

Fisher’s test was performed to compare the categorical data between IOPN and IPMN/IPNB and between pure IOPN and combined IOPN. Mann-Whitney’s U test was performed for comparing the sequential data. A *P*-value of <0.05 was considered statistically significant. JMP 13.2.1 statistical software (SAS Institute, Incorporation, Cary, NC) was used for the analyses. *P*-values were calculated using Student’s *t*-test for the results of digital gene expression analyses using NanoString.

## Supplementary information

Supplementary information.
