# Supplementary material for: Upregulation of follistatin and low apoptotic activity in intraductal oncocytic papillary neoplasm of the pancreatobiliary system
Source: Sci Rep. 2020 May 18;10:8179. doi: 10.1038/s41598-020-64920-6 (PMC7235027; doi:10.1038/s41598-020-64920-6)
Supplement: Supplementary file 1 — Supplementary information. [file 41598_2020_64920_MOESM1_ESM.docx]

Upregulation of follistatin and low apoptotic activity in intraductal oncocytic papillary neoplasm of the pancreatobiliary system

Supplementary Material

Jun Nakahodo^1^, MD; Yuki Fukumura^1^, MD, phD, MS; Tsuyoshi Saito^1^, MD, phD; Kenichi Hirabayashi^2^, MD, phD; Reiko Doi^1^, MD; Takuo Hayashi^1^, MD, phD; Takashi Yao^1,^ MD, phD

^1^ Department of Human Pathology, Juntendo University School of Medicine

^2^ Department of Pathology, Tokai University School of Medicine

**Corresponding Author:**  Yuki Fukumura

E-mail: [yfuku@juntendo.ac.jp](mailto:yfuku@juntendo.ac.jp)

Affiliation: Department of Human Pathology, Juntendo University School of Medicine

Address: Building A 10F, Hongo 2-1-1, Bunkyo-ku, Tokyo, 113-8421, Japan

Telephone: +81-3-5802-1037

Facsimile: +81-3-3812-1056

**Tables**

| **Supplementary Table 1. Cases Used in Multiple Gene Expression Analysis** | | | | | | | |
| --- | --- | --- | --- | --- | --- | --- | --- |
|  |  |  |  |  |  |  |  |
| **Tumor Group^a^** | **Case** | **Age** | **Sex** | **Tumor Site^b^** | **Histological Grade** | **Pure/Combined** | **Tumor Size(max)** |
| p-IOPN | O-12 | 69 | F | Head | high grade with invasion | Pure | 30 |
| p-IOPN | O-17 | 82 | F | Body to Tail | high grade with invasion | Combined | 25 |
| b-IOPN | O-19 | 73 | M | IH/Left | high grade | Pure | 30 |
| b-IOPN | O-20 | 76 | M | IH/Right | high grade | Pure | 37 |
| g-IPMN | P-1 | 48 | F | Body to Tail | low grade | NA | 55 |
| g-IPMN | P-2 | 61 | M | Body to Tail | low grade | NA | 18 |
| i-IPMN | P-14 | 77 | M | Head | high grade | NA | 75 |
| i-IPNB | B-1 | 78 | M | PH | high grade | NA | 25 |
| g-IPNB | B-2 | 66 | F | IH/Left | high grade | NA | 15 |
| g-IPNB | B-3 | 71 | F | IH/Right | high grade | NA | 65 |
| ^a^For Tumor Group, p-IOPN, IOPN of the pancreas; b-IOPN, IOPN of the bile duct; g/i-IPMN, IPMN of gastric/intestinal subtype; g/i-IPNB, IPNB of gastric/intestinal subtype | | | | | | | |
| ^b^For Tumor Site, IH, intrahepatic bile duct; PH, perihilar bile duct | | | | | | | |

| **Supplementary Table 2. Cases Usd in qPCR for FST and TGF-β1** | | | | | | | | | | |
| --- | --- | --- | --- | --- | --- | --- | --- | --- | --- | --- |
| **Tumor Group^a^** | **Case** | **Age** | **Sex** | **Tumor Site^b^** | **Histological Grade** | **Coexisted subtype** | **Size(max)** | **FST^c^** | **TGFB1^c^** |  |
| g-IPMN | P-1 | 48 | F | BT | low grade | NA | 55 |  |  |  |
| g-IPMN | P-2 | 61 | M | BT | low grade | NA | 18 | NA |  |  |
| g-IPMN | P-3 | 72 | M | H | low grade | NA | 38 |  |  |  |
| g-IPMN | P-4 | 73 | M | H | low grade | NA | 25 |  |  |  |
| g-IPMN | P-5 | 76 | F | H | high grade | NA | 60 |  |  |  |
| g-IPMN | P-6 | 77 | M | BT | high grade with invasion | NA | 75 |  |  |  |
| g-IPMN | P-7 | 78 | M | H | high grade | NA | 71 |  |  |  |
| g-IPMN | P-8 | 81 | F | H | high grade | NA | 30 |  |  |  |
| i-IPMN | P-9 | 52 | M | BT | high grade with invasion | NA | 27 |  |  |  |
| i-IPMN | P-10 | 68 | M | H | high grade with invasion | NA | 25 | NA |  |  |
| i-IPMN | P-11 | 70 | M | BT | high grade with invasion | NA | 22 |  |  |  |
| i-IPMN | P-12 | 72 | M | H | high grade | NA | 23 |  |  |  |
| i-IPMN | P-13 | 74 | F | BT | high grade | NA | 65 |  |  |  |
| i-IPMN | P-14 | 77 | M | H | high grade | NA | 75 |  |  |  |
| p-IOPN | O-2 | 45 | M | H | high grade with invasion | Pure | 15 | NA | NA |  |
| p-IOPN | O-9 | 64 | M | BT | high grade | Combined | 20 |  |  |  |
| p-IOPN | O-12 | 69 | F | H | high grade with invasion | Combined | 30 |  |  |  |
| p-IOPN | O-11 | 69 | F | H | high grade | Pure | 30 |  |  |  |
| p-IOPN | O-17 | 82 | F | BT | high grade with invasion | Pure | 25 |  |  |  |
| b-IOPN | O-19 | 73 | M | IH/Left | high grade | Pure | 30 |  |  |  |
| b-IOPN | O-20 | 76 | M | IH/Left | high grade | Pure | 37 |  |  |  |
| i-IPNB | B-1 | 78 | M | PH | high grade | NA | 25 |  |  |  |
| i-IPNB | B-4 | 79 | F | IH/Right | high grade | NA | 24 |  |  |  |
| g-IPNB | B-2 | 66 | F | IH/Left | high grade | NA | 15 |  |  |  |
| ^a^For Tumor Group, p-IOPN, IOPN of the pancreas; b-IOPN, IOPN of the bile duct; g/i-IPMN, IPMN of gastric/intestinal subtype; g/i-IPNB, IPNB of gastric/intestinal subtype | | | | | | | | | | |
| ^b^For Tumor Site, BT, body to tail of pacreas; H, head of pancreas, IH, intrahepatic bile duct; PH, perihilar bile duct | | | | | | | | | | |
| ^c^For FST/TGF-β1, NA stands for no amplification of cDNA | | | | | | | | | | |

| **Supplementary Table 3. Probes Used for qPCR (purchased from Applied Biosystems)** | | | |
| --- | --- | --- | --- |
| **Gene** | **Gene Symbol** | **Length of Amplicon** | **Assay ID** |
| actin beta | ACTB | 63 | Hs01060665_g1 |
| follistatin | FST | 61 | Hs01121165_g1 |
| transforming growth factor beta 1 | TGFB1 | 57 | Hs00998133_m1 |

| **Supplementary Table 4. Antibodies Used in this study** | | | | | | | |
| --- | --- | --- | --- | --- | --- | --- | --- |
| **Antibody** |  | **Clone** | **Buffer** | **Pretreatment** | **Dilution** | **Source** |  |
| MUC1 | monoclonal | Ma695 | CB | HIER | 1:0 | Leica Biosystems, Newcastle upon Tyne, UK |  |
| MUC2 | monoclonal | Ccp58 | CB | HIER | 1:1 | Leica Biosystems, Newcastle upon Tyne, UK |  |
| MUC5AC | monoclonal | CLH2 | CB | HIER | 1:500 | Abcam plc, Cambridge, UK |  |
| MUC6 | monoclonal | MUC6/916 | CB | HIER | 1:100 | Abcam plc, Cambridge, UK |  |
| Human Hepatocyte | monoclonal | OCH1E5 | TE | HIER | 1:100 | DAKO, Denmark |  |
| Mitochondria | monoclonal | 113-1 | CB | HIER | 1:1000 | Abcam plc, Cambridge, UK |  |
| Ki-67 | monoclonal | MIB-1 | CB | HIER | 1:200 | DAKO, Denmark |  |
| Follistatin (FST) | monoclonal | 85918 | CB | HIER | 1:25 | R&D Systems, Minneapolis, MN, USA |  |
| Cleaved Caspase-3 | polyclonal | Asp175 | CB | HIER | 1:400 | CST (MA, USA) |  |
| HIER, Heat-induced epitope retrieval; MUC, Mucin | | | | | | | |

| **Supplementary Table 5. Clinicopathological summary of IOPN cases** | | | | | | | | | | | | | |
| --- | --- | --- | --- | --- | --- | --- | --- | --- | --- | --- | --- | --- | --- |
| **Tumor Group^a^** | **Case** | **Institution^b^** | **Tumor Site^a^** | **Age** | **Sex** | **Tumor Size(max)** | **Duct Type** | **T factor** | **N factor** | **M factor** | **pStage**  **(UICC 8th)** | **Type** | **Coexisted Subtype** |
| p-IOPN | O-1 | Y | BT | 44 | M | 100 | Mixed | T1a | 0 | 0 | ⅠA | Pure | None |
| p-IOPN | O-2 | X | H | 45 | M | 15 | Mixed | T1a | 0 | 0 | ⅠA | Pure | None |
| p-IOPN | O-3 | Y | BT | 47 | F | 40 | Mixed | T1a | 0 | 0 | ⅠA | Pure | None |
| p-IOPN | O-4 | Y | H | 58 | F | 30 | Mixed | T1a | 0 | 0 | ⅠA | Pure | None |
| p-IOPN | O-5 | X | H | 59 | F | 30 | Mixed | Tis | 0 | 0 | 0 | Combined | Gastric |
| p-IOPN | O-6 | X | BT | 61 | F | 25 | Branch | Tis | 0 | 0 | 0 | Pure | None |
| p-IOPN | O-7 | Y | H | 61 | M | 35 | Branch | Tis | 0 | 0 | 0 | Pure | None |
| p-IOPN | O-8 | Y | H | 62 | M | 75 | Branch | Tis | 0 | 0 | 0 | Pure | None |
| p-IOPN | O-9 | X | BT | 64 | M | 12 | Mixed | Tis | 0 | 0 | 0 | Combined | Intestinal |
| p-IOPN | O-10 | Y | H | 67 | M | 30 | Branch | T1a | 0 | 0 | ⅠA | Pure | None |
| p-IOPN | O-11 | X | H | 69 | F | 22 | Mixed | Tis | 0 | 0 | 0 | Pure | None |
| p-IOPN | O-12 | X | H | 69 | F | 30 | Branch | T1a | 0 | 0 | ⅠA | Combined | Gastric |
| p-IOPN | O-13 | Y | BT | 75 | M | 50 | Mixed | T3 | 1 | 0 | ⅡB | Combined | Gastric |
| p-IOPN | O-14 | Y | H | 76 | F | 70 | Branch | T1a | 0 | 0 | ⅠA | Combined | Intestinal |
| p-IOPN | O-15 | Y | BT | 79 | F | 50 | Mixed | Tis | 0 | 0 | 0 | Pure | None |
| p-IOPN | O-16 | X | H | 80 | M | 22 | Mixed | Tis | 0 | 0 | 0 | Pure | None |
| p-IOPN | O-17 | X | BT | 82 | F | 25 | Branch | T1a | 0 | 0 | ⅠA | Pure | None |
| p-IOPN | O-18 | Y | BT | 82 | M | 26 | Mixed | Tis | 0 | 0 | 0 | Combined | Gastric |
| b-IOPN | O-19 | X | IH/Left | 73 | M | 30 | ― | Tis | 0 | 0 | 0 | Pure | None |
| b-IOPN | O-20 | X | IH/Right | 76 | M | 37 | ― | Tis | 0 | 0 | 0 | Pure | None |
| b-IOPN | O-21 | Y | IH/Left | 79 | M | 25 | ― | T1a | 0 | 0 | I A | Pure | None |
| b-IOPN | O-22 | X | IH/Left | 61 | M | 65 | ― | Tis | 0 | 0 | I | Combined | Gastric |
| ^a^ For Tumor Group, p-/b-/IOPN, IOPN of pancreas/bile duct; ^b^ For Instutution, X/Y, the first author's/forth author's insitution | | | | | | | | | | | | | |
| ^c^ For Tumor Site, BT, body to tail of pacreas; H, head of pancreas, IH, intrahepatic bile duct | | | | | | | | | | | | | |

| **Supplementary Table 6. Differently expressed genes between IOPN and gastric IPMN/IPNB** | | | | | | | | | |  |
| --- | --- | --- | --- | --- | --- | --- | --- | --- | --- | --- |
| The top 20 genes with the lowest p-value were listed. | | | | | | | | | |  |
| **gene** | **fold change (log2)** | **Lower confidence limit (log2)** | **Upper confidence limit (log2)** | **Linear fold change** | **Lower confidence limit (linear)** | **Upper confidence limit (linear)** | **P-value** | **adjusted p.value** | **attributed pathway** | |
| FST | 5.34 | 4.35 | 6.32 | 40.40 | 20.40 | 79.90 | 1.43E-05 | 0.047 | TGF-beta | |
| NUPR1 | -3.39 | -4.08 | -2.70 | 0.10 | 0.06 | 0.15 | 2.69E-05 | 0.047 | Transcriptional Misregulation | |
| IDH2 | 2.12 | 1.65 | 2.58 | 4.33 | 3.13 | 6.00 | 4.77E-05 | 0.047 | Driver Gene | |
| PIM1 | -2.20 | -2.70 | -1.70 | 0.22 | 0.15 | 0.31 | 5.42E-05 | 0.047 | JAK-STAT | |
| PROM1 | -4.15 | -5.10 | -3.19 | 0.06 | 0.03 | 0.11 | 6.03E-05 | 0.047 | Transcriptional Misregulation | |
| E2F1 | 3.34 | 2.57 | 4.11 | 10.10 | 5.92 | 17.30 | 6.35E-05 | 0.047 | Cell Cycle - Apoptosis | |
| MMP7 | -6.06 | -7.50 | -4.62 | 0.02 | 0.01 | 0.04 | 7.53E-05 | 0.048 | Wnt | |
| PRKAR2A | 0.92 | 0.69 | 1.14 | 1.89 | 1.61 | 2.21 | 9.43E-05 | 0.053 | Cell Cycle - Apoptosis | |
| MAP3K14 | -1.49 | -1.90 | -1.09 | 0.36 | 0.27 | 0.47 | 0.00017 | 0.077 | Cell Cycle - Apoptosis, MAPK | |
| EGFR | 1.93 | 1.40 | 2.45 | 3.80 | 2.65 | 5.45 | 0.00017 | 0.077 | Driver Gene, MAPK, PI3K, Ras | |
| PRKAR1B | 1.30 | 0.92 | 1.68 | 2.46 | 1.89 | 3.20 | 0.00027 | 0.104 | Cell Cycle - Apoptosis | |
| LEFTY1 | -6.61 | -8.56 | -4.66 | 0.01 | 0.00 | 0.04 | 0.00029 | 0.104 | TGF-beta | |
| CDC25A | 3.74 | 2.63 | 4.85 | 13.40 | 6.20 | 28.80 | 0.00030 | 0.104 | Cell Cycle - Apoptosis | |
| GRIA3 | -2.91 | -3.78 | -2.04 | 0.13 | 0.07 | 0.24 | 0.00033 | 0.104 | Transcriptional Misregulation | |
| PML | -1.09 | -1.43 | -0.76 | 0.47 | 0.37 | 0.59 | 0.00038 | 0.105 | Transcriptional Misregulation | |
| HRAS | 1.37 | 0.95 | 1.79 | 2.59 | 1.93 | 3.47 | 0.00039 | 0.105 | Driver Gene, MAPK, PI3K, Ras | |
| EP300 | -0.63 | -0.82 | -0.43 | 0.65 | 0.57 | 0.74 | 4.00E-04 | 0.105 | Cell Cycle - Apoptosis, Driver Gene, JAK-STAT, Notch, TGF-beta, Wnt | |
| TLR4 | -1.17 | -1.53 | -0.80 | 0.45 | 0.35 | 0.58 | 0.00043 | 0.107 | PI3K | |
| IL6R | -2.41 | -3.19 | -1.63 | 0.19 | 0.11 | 0.32 | 0.00053 | 0.125 | JAK-STAT, PI3K | |
| DUSP5 | -2.28 | -3.03 | -1.52 | 0.21 | 0.12 | 0.35 | 0.00059 | 0.130 | MAPK | |

| **Supplementary Table 7. Differently expressed genes between IOPN and intestinal IPMN/IPNB** | | | | | | | | | |
| --- | --- | --- | --- | --- | --- | --- | --- | --- | --- |
| The top 20 genes with the lowest p-value were listed. | | | | | | | | | |
| **gene** | **fold change (log2)** | **Lower confidence limit (log2)** | **Upper confidence limit (log2)** | **Linear fold change** | **Lower confidence limit (linear)** | **Upper confidence limit (linear)** | **P-value** | **adjusted p.value** | **attributed pathway** |
| SMAD9 | -5.99 | -6.66 | -5.31 | 0.02 | 0.01 | 0.03 | 5.05E-07 | 0.002 | TGF-beta |
| SGK2 | 4.38 | 3.50 | 5.26 | 20.80 | 11.30 | 38.20 | 2.45E-05 | 0.040 | PI3K |
| FST | 5.81 | 4.59 | 7.02 | 56.00 | 24.10 | 130.00 | 3.27E-05 | 0.040 | TGF-beta |
| MFNG | -2.99 | -3.62 | -2.35 | 0.13 | 0.08 | 0.20 | 3.60E-05 | 0.040 | Notch |
| TLR4 | -1.86 | -2.30 | -1.42 | 0.28 | 0.20 | 0.37 | 7.05E-05 | 0.053 | PI3K |
| PROM1 | -4.93 | -6.10 | -3.77 | 0.03 | 0.01 | 0.07 | 7.06E-05 | 0.053 | Transcriptional Misregulation |
| ARNT2 | -4.76 | -6.00 | -3.52 | 0.04 | 0.02 | 0.09 | 0.00013 | 0.085 | Transcriptional Misregulation |
| PRKAR2A | 0.94 | 0.66 | 1.21 | 1.91 | 1.58 | 2.32 | 0.00030 | 0.166 | Cell Cycle - Apoptosis |
| EGFR | 2.12 | 1.48 | 2.76 | 4.36 | 2.79 | 6.79 | 0.00033 | 0.166 | Driver Gene, MAPK, PI3K, Ras |
| ZAK | -1.36 | -1.79 | -0.92 | 0.39 | 0.29 | 0.53 | 0.00047 | 0.211 | MAPK |
| MAPK3 | -1.70 | -2.27 | -1.14 | 0.31 | 0.21 | 0.45 | 0.00059 | 0.239 | MAPK, PI3K, Ras, TGF-beta |
| IDH2 | 1.65 | 1.08 | 2.23 | 3.15 | 2.12 | 4.69 | 0.00077 | 0.280 | Driver Gene |
| CDC25B | -2.74 | -3.72 | -1.76 | 0.15 | 0.08 | 0.30 | 0.00091 | 0.280 | Cell Cycle - Apoptosis, MAPK |
| IL23R | -1.98 | -2.69 | -1.26 | 0.25 | 0.16 | 0.42 | 0.00098 | 0.280 | JAK-STAT |
| HRAS | 1.45 | 0.93 | 1.97 | 2.73 | 1.90 | 3.92 | 0.00098 | 0.280 | Driver Gene, MAPK, PI3K, Ras |
| BID | -1.32 | -1.80 | -0.84 | 0.40 | 0.29 | 0.56 | 0.00100 | 0.280 | Cell Cycle - Apoptosis |
| HES5 | -1.68 | -2.31 | -1.06 | 0.31 | 0.20 | 0.48 | 0.00117 | 0.306 | Notch |
| RB1 | 0.99 | 0.61 | 1.37 | 1.99 | 1.52 | 2.58 | 0.00141 | 0.306 | Cell Cycle - Apoptosis, Driver Gene |
| FGF10 | -5.19 | -7.20 | -3.17 | 0.03 | 0.01 | 0.11 | 0.00149 | 0.306 | MAPK, PI3K, Ras |
| CACNB2 | -4.54 | -6.30 | -2.77 | 0.04 | 0.01 | 0.15 | 0.00151 | 0.306 | MAPK |

| **Supplementary Table 8. Differently expressed genes between IOPN of the pancreas and bile duct** | | | | | | | | | |  |
| --- | --- | --- | --- | --- | --- | --- | --- | --- | --- | --- |
| The top 20 genes with the lowest p-value were listed. | | | | | | | | | |  |
| **gene** | **fold change (log2)** | **Lower confidence limit (log2)** | **Upper confidence limit (log2)** | **Linear fold change** | **Lower confidence limit (linear)** | **Upper confidence limit (linear)** | **P-value** | **adjusted p.value** | **attributed pathway** | |
| PAX8 | -3.18 | -3.44 | -2.92 | 0.11 | 0.09 | 0.13 | 0.00016 | 0.703 | Transcriptional Misregulation | |
| BID | -0.74 | -0.85 | -0.62 | 0.60 | 0.56 | 0.65 | 0.00106 | 1.000 | Cell Cycle - Apoptosis | |
| PBX3 | -1.43 | -1.71 | -1.15 | 0.37 | 0.31 | 0.45 | 0.00210 | 1.000 | Transcriptional Misregulation | |
| BMP5 | -4.00 | -4.81 | -3.18 | 0.06 | 0.04 | 0.11 | 0.00242 | 1.000 | TGF-beta | |
| CACNB4 | -4.22 | -5.09 | -3.35 | 0.05 | 0.03 | 0.10 | 0.00244 | 1.000 | MAPK | |
| NR4A1 | -2.81 | -3.38 | -2.23 | 0.14 | 0.10 | 0.21 | 0.00249 | 1.000 | MAPK, PI3K | |
| KITLG | -1.73 | -2.10 | -1.36 | 0.30 | 0.23 | 0.39 | 0.00272 | 1.000 | PI3K, Ras | |
| HSP90B1 | -1.58 | -1.98 | -1.19 | 0.33 | 0.25 | 0.44 | 0.00418 | 1.000 | PI3K | |
| MET | 1.56 | 1.17 | 1.95 | 2.95 | 2.25 | 3.86 | 0.00427 | 1.000 | Driver Gene, PI3K, Ras, Transcriptional Misregulation | |
| SFRP4 | 2.69 | 2.00 | 3.37 | 6.44 | 4.00 | 10.40 | 0.00460 | 1.000 | Wnt | |
| NSD1 | 1.16 | 0.85 | 1.48 | 2.24 | 1.80 | 2.80 | 0.00563 | 1.000 | Chromatin Modification | |
| CLCF1 | 1.68 | 1.21 | 2.16 | 3.21 | 2.31 | 4.47 | 0.00614 | 1.000 | JAK-STAT | |
| ID2 | -1.05 | -1.36 | -0.75 | 0.48 | 0.39 | 0.60 | 0.00652 | 1.000 | TGF-beta, Transcriptional Misregulation | |
| GADD45G | -1.86 | -2.40 | -1.32 | 0.28 | 0.19 | 0.40 | 0.00656 | 1.000 | Cell Cycle - Apoptosis, MAPK | |
| MPO | -1.28 | -1.65 | -0.91 | 0.41 | 0.32 | 0.53 | 0.00661 | 1.000 | Transcriptional Misregulation | |
| NR4A3 | -3.44 | -4.46 | -2.42 | 0.09 | 0.05 | 0.19 | 0.00707 | 1.000 | Transcriptional Misregulation | |
| THBS4 | 7.15 | 5.02 | 9.29 | 142.00 | 32.40 | 626.00 | 0.00718 | 1.000 | PI3K | |
| MAP2K6 | 2.31 | 1.62 | 3.00 | 4.96 | 3.07 | 8.01 | 0.00727 | 1.000 | MAPK | |
| IL22RA2 | -3.29 | -4.34 | -2.25 | 0.10 | 0.05 | 0.21 | 0.00855 | 1.000 | JAK-STAT | |
| RXRG | -2.04 | -2.69 | -1.39 | 0.24 | 0.16 | 0.38 | 0.00868 | 1.000 | Transcriptional Misregulation | |

|  | **Supplementary Table 9. Immunohistochemical results** | | | | | | | | | | | | | | | | | | |
| --- | --- | --- | --- | --- | --- | --- | --- | --- | --- | --- | --- | --- | --- | --- | --- | --- | --- | --- | --- |
|  | |  |  | **IOPN** | | | | |  |  | |  | **IPMN/IPNB** | | | | |  | |
|  | | **IOPN**  **(p+b)** |  | **p-IOPN** | |  | **b-IOPN** | | **Pure vs. combined P value** |  | **IPMN + IPNB** |  | **IPMN** | |  | **IPNB** | |  | **IOPN vs. IPMN/IPNB P value** |
|  | |  |  | **pure** | **combined** |  | **pure** | **combined** |  |  |  |  | **Gastric**  **type** | **Intestinal type** |  | **Gastric**  **type** | **Intestinal type** |  |  |
| **Antibody^φ^** | | **(N=22)** |  | **(N=12)** | **(N=6)** |  | **(N=3)** | **(N=1)** |  |  | **(N=69)** |  | **(N=41)** | **(N=20)** |  | **(N=2)** | **(N=2)** |  |  |
| **MUC1** | | 11/9/2 |  | 6/5/1 | 3/2/1 |  | 2/1/0 | 0/1/0 | 0.81 |  | 56/10/3 |  | 34/6/1 | 19/0/1 |  | 0/3/1 | 3/1/0 |  | 0.015 |
| **MUC2** | | 13/9/0 |  | 6/6/0 | 4/2/0 |  | 3/0/0 | 0/1/0 | 0.899 |  | 31/17/21 |  | 26/11/4 | 1/4/15 |  | 4/0/0 | 0/2/2 |  | 0.012 |
| **MUC5AC** | | 0/2/20 |  | 0/0/12 | 0/1/5 |  | 0/1/2 | 0/0/1 | 0.563 |  | 1/12/56 |  | 0/4/37 | 0/3/17 |  | 1/2/1 | 0/3/1 |  | 0.534 |
| **MUC6** | | 1/2/19 |  | 1/0/11 | 0/2/4 |  | 0/1/2 | 0/0/1 | 0.680 |  | 14/34/21 |  | 2/20/19 | 9/10/1 |  | 0/4/0 | 3/0/1 |  | ＜0.0001 |
| **Hepatocyte** | | 3/1/18 |  | 0/0/12 | 3/1/2 |  | 0/0/3 | 0/0/1 | 0.005 |  | NP |  | NP | NP |  | NP | NP |  | － |
| **Mitochondria** | | 0/4/18 |  | 0/1/11 | 0/3/3 |  | 0/0/3 | 0/0/1 | 0.040 |  | NP |  | NP | NP |  | NP | NP |  | － |
| **Ki-67 mean, % (range)** | | 17.9%  (5-50) |  | 15.0%  (5-30) | 27.0%  (5-50) |  | 18.0%  (10-25) | 10.0% | 0.212 |  | 21.0%  (2-85) |  | 12.8%  (2-39) | 40.9%  (7-85) |  | 14.0%  (8-20) | 13.3%  (3-25) |  | 0.530 |
| **Follistatin** | | 0/7/15 |  | 0/1/11 | 0/4/2 |  | 0/1/2 | 0/1/0 | 0.005 |  | 29/33/7 |  | 18/20/3 | 8/10/2 |  | 1/2/1 | 2/1/1 |  | ＜0.0001 |
| **Cleaved Caspase 3** | | 6/12/4 |  | 3/8/1 | 2/1/3 |  | 1/2/0 | 0/1/0 | 0.094 |  | 19/13/37 |  | 15/9/17 | 2/3/15 |  | 2/1/1 | 0/0/4 |  | 0.002 |

**^φ^** For MUC1,MUC2, MUC5AC, MUC6, Hepatocyte, and Mitochondria, 0, no positive cells; 1 <50% of tumor cells are positive; 2≧50% of tumor cells are positive; NP, not peformed

For Ki-67 LI, MIB-1 positive cells/total tumor cells were recorded with percentage

For CC3, 0, no positive cells; 1, positive cells were only scatteredly seen; 2, positive cells were in aggregates.

For FST, 0, negative for tumor cells; 1, weakly positive; 2, intensely positive


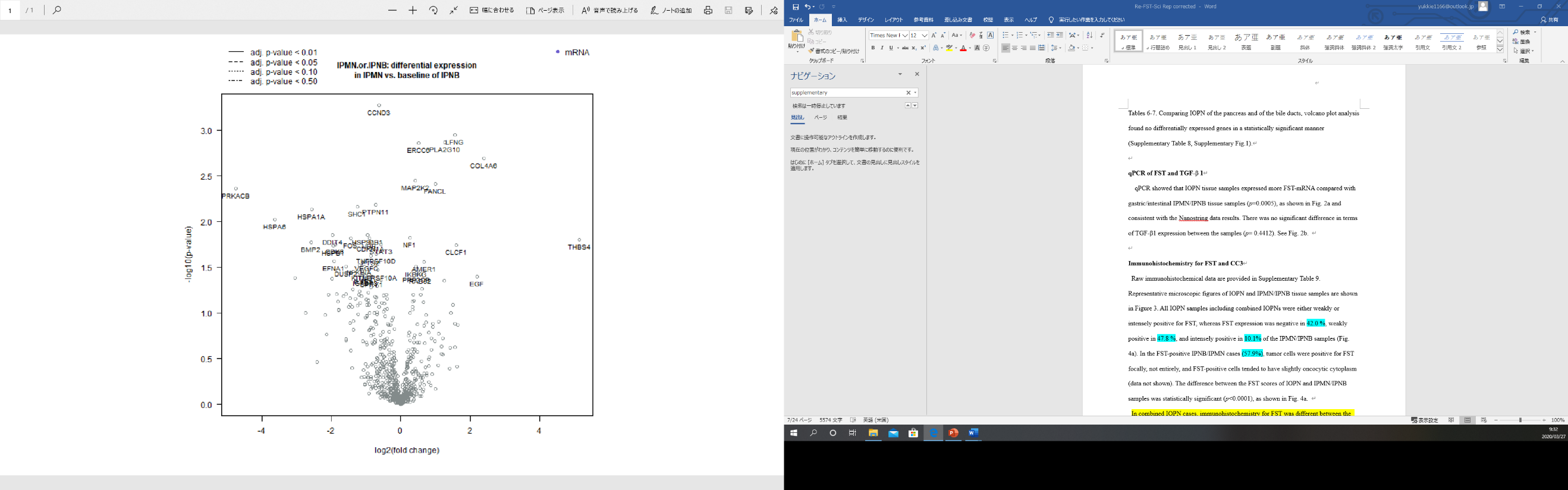
Supplementary Figure 1. Volcano plot between IOPN of pancreas and of the bile duct

Volcano plot analysis found no statistically differentially expressed genes between IOPN of pancreas and that of the bile duct.
